# Real-world clinical outcomes and economic burden of discontinuation of taxane therapy among patients with metastatic castration-resistant prostate cancer

**DOI:** 10.1007/s10147-026-03102-2

**Published:** 2026-06-25

**Authors:** Neal Shore, Jennifer Nguyen, Barinder Kang, Amrita Sawhney, Clare Byrne, Jackson Tang, Magdaliz Gorritz, Chi-Chang Chen, Jeetvan Patel

**Affiliations:** 1 START Carolinas/Carolina Urologic Research Center, Myrtle Beach, SC USA; 2 Novartis Pharmaceuticals Corporation, East Hanover, NJ USA; 3 Asclepius Analytics, New York, NY USA; 4 IQVIA, Wayne, PA USA

**Keywords:** Prostate cancer, Castration resistance, Taxane-based chemotherapy, Metastatic, Cost

## Abstract

**Background:**

Metastatic castration-resistant prostate cancer (mCRPC) treatment commonly includes first-line (1L) androgen receptor pathway inhibitors (ARPIs) followed by second-line (2L) taxane-based chemotherapy. Adverse events (AEs) may lead to early taxane discontinuation, which can negatively affect outcomes and costs. This retrospective observational study assessed real-world outcomes, AEs, and costs of 2L taxane therapy by number of cycles among patients with mCRPC previously treated with a 1L ARPI; outcomes were compared with patients receiving 2L ARPI.

**Methods:**

Overall survival (OS; overall and by number of taxane cycles received) was determined for 2L taxane and compared with 2L ARPI using the Flatiron Health electronic health record database (Jul 1, 2012, to Jun 30, 2020). Taxane-related AEs and associated costs were estimated using the IQVIA PharMetrics® Plus claims database (Jan 1, 2013, to Aug 31, 2021).

**Results:**

Overall, 2L ARPI (*n* = 473) vs. taxane (*n* = 214) was associated with longer median OS (15.4 vs. 13.6 months). Patients receiving > 8 (*n* = 48) vs. ≤ 8 taxane cycles (*n* = 166) had longer median OS (18.4 vs. 11.9 months; *p* = 0.006). Patients receiving > 8 vs. ≤ 8 taxane cycles were also more likely to experience an AE (95% vs. 88%) but had lower mean AE-related total healthcare costs per patient per month ($3,429 vs. $6,334).

**Conclusion:**

Patients who received > 8 cycles of 2L taxane had longer observed survival. However, these results are subject to selection bias and immortal time bias. Prospective or time-adjusted analyses are needed.

**Supplementary Information:**

The online version contains supplementary material available at https://doi.org/10.1007/s10147-026-03102-2.

## Introduction

Prostate cancer (PC) is the second most common cancer in men, with > 1.4 million new cases per year globally and > 300,000 new cases per year in the US [1–3]. Patients with metastatic castration-resistant PC (mCRPC) have a poor prognosis, rapid disease progression and high mortality [4, 5].

Effective available therapies include androgen receptor pathway inhibitors (ARPIs), taxanes, immunotherapies, radiopharmaceuticals, and poly (ADP-ribose) polymerase inhibitors [6–20]. The US National Comprehensive Cancer Network® (NCCN®) Clinical Practice Guidelines in Oncology (NCCN Guidelines®) recommends the ARPIs abiraterone and enzalutamide and the taxane docetaxel for first-line (1L) mCRPC treatment [21]. Second line (2L) options depend on whether patients have previously received ARPIs or taxanes, with the NCCN Guidelines® recommending in each case that patients switch to a treatment class with a different mechanism of action [21]. Given the availability of numerous treatment options and the lack of head-to-head comparison trials, selecting and sequencing treatment options is challenging. Prior real-world studies have suggested that, despite guidelines, ARPIs are the most common 1L and 2L treatment choice for patients with metastatic prostate cancer, including mCRPC [22–24].

Taxane use is limited by adverse events (AEs) [25] that can lead to early discontinuation. Although previous studies suggest that patients should receive 10 cycles of taxane [26–29], prior real-world evidence indicates that the number of cycles patients receive varies widely, with many receiving considerably fewer than 10 cycles [9, 27–31]. Whether early treatment discontinuation affects patient outcomes is currently unclear.

The aims of this study were to determine the proportion of patients who discontinue 2L taxane therapy early and to examine whether there is an association between early discontinuation and clinical outcomes, AEs, or costs associated with AEs. The threshold for “early discontinuation” was defined as receipt of fewer taxane cycles than the number found to be associated with a similar survival trajectory to that of 2L ARPIs. This was achieved by determining the number of cycles of taxanes patients received as 2L therapy in the real-world setting and assessing the clinical outcomes by number of cycles compared with patients who received ARPIs as 2L therapy.

## Patients and methods

### Data sources

This retrospective cohort study utilized the US Flatiron Health electronic health record (EHR) database (Jul 1, 2012, to Jun 30, 2020) to assess clinical effectiveness; and the US IQVIA PharMetrics® Plus claims database (Jan 1, 2013, to Aug 31, 2021) to assess safety and economic outcomes. The longitudinal Flatiron Health database features quality-controlled, de-identified patient-level demographic, treatment, and mortality data from over 280 cancer clinics (~ 800 care sites) spanning more than 2.2 million patients with cancer. The IQVIA PharMetrics Plus de-identified database features adjudicated claims from over 190 million unique enrollees, including more than 61 million people with ≥ 3 years of continuous enrollment.

### Patient selection

Men diagnosed with PC (*International Classification of Diseases* 9th/10th Revision, Clinical Modification [ICD-9/10-CM] 185.x/C61.x) and with documented dates of diagnosis for both metastatic disease and CRPC were included. Each patient was assigned an mCRPC diagnosis date, defined as the latter of the dates of CRPC diagnosis and metastatic PC diagnosis (in the effectiveness analyses), or the date of the first claim for an mCRPC therapy (docetaxel, cabazitaxel, abiraterone, enzalutamide, radium-223, mitoxantrone, estramustine, sipuleucel-T) after castration (in the safety and economic analyses).

Patients must have received an ARPI as their 1L therapy post-mCRPC diagnosis and initiated 2L therapy with another ARPI or a taxane. The date of initiation of the 2L treatment was defined as the index date and had to occur between Jan 1, 2013, and Dec 31, 2019, for the effectiveness analyses and between Jan 1, 2014, and Jul 31, 2021, for the safety and economic analyses. For the effectiveness analyses, patients needed ≥ 2 clinic visits for inclusion, and for the safety and economic analyses, patients were required to have continuous enrollment for ≥ 12 months pre-index (baseline period) and 1-month post-index (follow-up period).

Patients in each database were divided into two groups based on their 2L treatment – ARPI (abiraterone or enzalutamide) vs. taxane (docetaxel or cabazitaxel). For the effectiveness analyses, patients were followed from the index date until the first occurrence of death, loss to follow-up, or the end of the study period. In the safety and economic analyses, patients were followed from the index date until the earliest of the end of 2L therapy, end of continuous enrollment, or end of the study period.

### Outcomes and measures

Patient characteristics were evaluated at index for both groups. Outcomes assessed over the follow-up period included number of taxane cycles received and duration of therapy in the taxane group, and overall survival (OS) and progression-free survival (PFS) in both groups. In the taxane group, OS was assessed by the number of taxane cycles patients received, and the threshold for early discontinuation was determined by identifying the number of cycles associated with the most similar survival trajectory when compared with 2L ARPIs. ‘Early discontinuers’ were defined as patients who stopped receiving 2L taxanes when they had received fewer than the number of cycles found to achieve outcomes at least comparable to those of ARPIs. Patients in the taxane group were divided according to this threshold and OS and PFS were compared between those who discontinued treatment early and those who did not. The incidence of chemotherapy-associated AEs (identified via ICD-9/10-CM diagnosis codes, treatments [National Drug Codes] and procedures [Current Procedural Terminology; CPT]) and AE-related healthcare costs during taxane treatment were also determined and compared between patients who discontinued early and those who did not. Endpoints are summarized per database in Supplemental Table 1.

**Table 1:** Sample selection: Flatiron health

Study population	*N*
Diagnosed with prostate cancer (ICD-9-CM: 185.x; ICD-10-CM: C61.x) and ≥ 18 years old at initiation of 1L therapy in the mCRPC setting	160,914
≥ 2 documented clinical encounters on different days in the Flatiron network on or after Jan 1, 2013	145,168
Male sex	144,825
Diagnosed with metastatic status on or after Jan 1, 2013, with histology confirmed as adenocarcinoma or NOS	14,925
Diagnosed with castration resistance^a^	2,902
Treated with ≥ 1 of the following therapies in the metastatic setting: enzalutamide, abiraterone, cabazitaxel, mitoxantrone, sipuleucel-T, docetaxel, radium-223^b^	2,902
Exclude patients with missing clinical outcomes (*n* = 92) or treatment information data (*n* = 14)^c^	2,796
Exclude patients with a secondary cancer diagnosis prior to prostate cancer diagnosis date (*n* = 147)^c^	2,649
Exclude patients treated with a clinical study drug as 1L therapy, either as monotherapy or in combination with any other therapy (*n* = 75)^c^	2,574
Exclude patients not treated with 1L ARPI therapy (enzalutamide, abiraterone) followed by 2L ARPI therapy (enzalutamide, abiraterone) or 2L taxane therapy (docetaxel, cabazitaxel) (*n* = 1,887)^c^	687
Final study population	687

^a^Castration resistance status was confirmed when a patient satisfied any of the following three conditions: 1) documentation of CRPC by the physician; 2) rising PSA level of ≥ 2 ng/mL after initial hormone therapy, followed by at least one further rise ≤ 3 months after the first increase; or 3) physician documentation of a rising PSA level or PSA progression on the 1L of hormone therapy, accompanied by a change in treatment. A patient was defined as having mCRPC at the later of the metastatic diagnosis date and the CRPC date

^b^Drugs designated in the Version 4.2024 NCCN Guidelines as the key recommended therapies indicated for patients with mCRPC [61]

^c^Across the analyses, patients were censored if they initiated a clinical study drug at any time. In addition, for PFS, patients were censored at the last clinic note date; for OS, censoring was based on the latest of the last clinic note date, last oral therapy start date, and last visit date

1L: first-line; 2L: second-line; ARPI: androgen receptor pathway inhibitor; CRPC: castration-resistant prostate cancer; ICD-9-CM: International Classification of Diseases, 9th Revision, Clinical Modification; ICD-10-CM: International Classification of Diseases, 10th Revision, Clinical Modification; mCRPC: metastatic castration-resistant prostate cancer; NCCN®: National Comprehensive Cancer Network®; NOS: not otherwise specified; OS: overall survival; PFS: progression-free survival; PSA: prostate-specific antigen

### Statistical analysis

All patient characteristics and outcomes in both cohorts were summarized descriptively. Kaplan–Meier OS curves were constructed for the 2L taxane group, stratified by the total number of cycles received, and overlaid with curves for the 2L ARPI cohort to determine the number of taxane cycles corresponding to a comparable OS. This number was then used as a proxy for ARPI in the subsequent Cox modeling analyses, in which both the hazard ratio (HR) comparing the number of taxane cycles with ARPI that was closest to 1, and the median OS closest to ARPI were determined to inform the definition of the threshold for early discontinuation. In addition, the comparative effect of each number of taxane cycles versus that of the proxy ARPI was evaluated using a spline curve to estimate the HR for each cycle.

Next, OS and PFS curves were constructed for the 2L taxane cohort stratified by whether patients did or did not discontinue treatment early and compared using the log rank test. All survival analyses were performed unadjusted and repeated using inverse probability of treatment weighting (IPTW) to adjust for all available patient characteristic variables.

AEs were compared between patients who discontinued taxanes early and those who did not using the Chi-square test. AE-related costs were reported on a per-patient-per-month (PPPM) basis; independent sample t-tests and Wilcoxon rank-sum tests were used to conduct comparisons of mean and median costs between the two groups. Further details are included in the Supplemental methods.

## Results

### Patient characteristics

A total of 687 patients were included from the Flatiron database and 354 patients from the PharMetrics database (Tables 1 and 2). Most patients received 2L ARPI (Flatiron: 473 of 687 [68.9%]; PharMetrics: 196 of 354 [55.4%]), with less than half receiving 2L taxane (Flatiron: 214 of 687 [31.1%; 186 docetaxel; 28 cabazitaxel]; PharMetrics: 158 of 354 [44.6%]).

**Table 2:** Sample selection: IQVIA PharMetrics Plus

Study population	*N*
Diagnosed with prostate cancer (ICD-9-CM: 185.x; ICD-10-CM: C61.x) based on ≥ 2 outpatient diagnoses (at least 30 days apart) or one inpatient diagnosis in any position during the index period	346,716
Diagnosed with metastasis (ICD-9-CM: 196.x, 197.x, 198.x; ICD-10-CM: C77.x, C78.x, C79.x) based on ≥ 2 outpatient diagnoses (at least 30 days apart) or one inpatient diagnosis in any position occurring on or after the initial prostate cancer diagnosis and during the index period	33,886
Medical or surgical castration^a^ either before or after diagnosis of metastasis	21,423
After date of castration, ≥ 1 claim for any of the following mCRPC therapies^b^: enzalutamide, abiraterone, cabazitaxel, mitoxantrone, estramustine, sipuleucel-T, docetaxel, and radium-223	13,036
Treatment with 1L ARPI therapy (enzalutamide, abiraterone) after castration^b^	8,701
Treatment with 2L ARPI therapy (enzalutamide, abiraterone) or 2L taxane therapy (docetaxel, cabazitaxel)^c^	1,426^d^
Male sex and ≥ 18 years old at initiation of 2L therapy for mCRPC	1,419
Continuous enrollment in PharMetrics Plus for ≥ 12 months pre-index and ≥ 1-month post-index	1,030
Exclude patients with multiple primary tumor types, except for non-malignant skin cancer, during the 12 months pre-index or any time after index	597
Exclude patients with data quality issues^e^	354
Final study population	354

^a^Medical castration was defined as having ≥ 1 claim for any anti-androgens, androgen-synthesis inhibitors, estrogens, or progestins AND one claim for any LHRH agonist OR having a claim with ICD-10-CM diagnosis code Z19.2. Surgical castration was defined as having ≥ 1 claim for surgical castration (diagnosis and medical procedure)

^b^Date of first claim for mCRPC therapy was considered the date of mCRPC diagnosis

^c^Patients were required to switch from 1L ARPI to a different 2L ARPI or taxane directly, with no other treatment lines in between

^d^Excludes patients who received both an ARPI and taxane as 1L therapy and had no switch in either the ARPI or taxane used in 2L therapy (*n* = 46). For example, a patient receiving 1L enzalutamide + docetaxel and then 2L leuprolide + docetaxel would have been excluded

^e^Specific data quality issues include: missing or invalid age, sex, or enrollment dates; Medicare Cost coverage, SCHIP, or age ≥ 65 years and not covered by Medicare Advantage

1L: first-line; 2L: second-line; ARPI: androgen receptor pathway inhibitor; ICD-9-CM: International Classification of Diseases, 9th Revision, Clinical Modification; ICD-10-CM: International Classification of Diseases, 10th Revision, Clinical Modification; LHRH: luteinizing hormone-releasing hormone; mCRPC: metastatic castration-resistant prostate cancer; SCHIP: State Children’s Health Insurance Program

Demographic and clinical characteristics in the 2L ARPI and taxane groups were broadly similar between groups (Tables 3 and 4).

**Table 3:** Patient characteristics: Flatiron health

	Treatment cohort		
	2L ARPI *N* = 473	2L taxane *N* = 214	*p* value^a^
Age, years, mean (SD)	74.0 (7.5)	70.5 (7.9)	< 0.0001
Race, *n* (%)			
White	292 (61.7)	153 (71.5)	0.13
Asian	14 (3.0)	3 (1.4)	
Black or African American	69 (14.6)	21 (9.8)	
Other	78 (16.5)	29 (13.6)	
Unknown	20 (4.2)	8 (3.7)	
Ethnicity, *n* (%)			
Hispanic or Latino	27 (5.7)	10 (4.7)	0.58
ECOG performance status, *n* (%)			
0	102 (31.7)	60 (36.8)	0.29
1	155 (48.1)	80 (49.1)	
2	51 (15.8)	20 (12.3)	
≥ 3	14 (4.3)	3 (1.8)	
Gleason score, *n* (%)			
> 7	218 (46.1)	111 (51.9)	0.35
≤ 7	99 (20.9)	38 (17.8)	
Unknown	156 (33.0)	65 (30.4)	
ALP, U/L, median (IQR)	88.5 (63.0–149.0)	88.0 (68.0–142.0)	0.46
Low,* n* (%)	8 (4.4)	4 (4.3)	0.99
Normal, *n* (%)	117 (64.3)	59 (63.4)	
Elevated, *n* (%)	57 (31.3)	30 (32.3)	
PSA, µg/L, median (IQR)	14.6 (2.7–61.1)	17.0 (4.6–74.5)	0.42
Low, *n* (%)	0 (0.0)	0 (0.0)	0.31
Normal, *n* (%)	52 (28.9)	21 (23.1)	
Elevated, *n* (%)	128 (71.1)	70 (76.9)	
Hb, g/dL, median (IQR)	12.6 (11.1–13.6)	12.5 (11.5–13.4)	0.70
Low,* n* (%)	109 (58.9)	59 (62.8)	0.66
Normal, *n* (%)	75 (40.5)	35 (37.2)	
Elevated, *n* (%)	1 (0.5)	0 (0.0)	
LDH, U/L, median (IQR)	216.0		
(177.5–264.0)	216.5		
(161.5–374.0)	0.98		
Low, *n* (%)	1 (1.9)	3 (15.0)	0.03
Normal, *n* (%)	40 (76.9)	10 (50.0)	
Elevated, *n* (%)	11 (21.2)	7 (35.0)	

^a^*p* values correspond to comparisons between the 1L ARPI/2L ARPI and 1L ARPI/2L taxane cohorts. Values in bold indicate statistical significance. 1L: first-line; 2L: second-line; ALP: alkaline phosphatase; ARPI: androgen receptor pathway inhibitor; ECOG: Eastern Cooperative Oncology Group; Hb: hemoglobin; IQR: interquartile range; LDH: lactate dehydrogenase; PSA: prostate-specific antigen; SD: standard deviation

**Table 4:** Patient characteristics: IQVIA PharMetrics Plus

	Treatment cohort		
	2L ARPI *N* = 196	2L taxane *N* = 158	*p* value^a^
Age, years			
Mean (SD)	66.9 (9.7)	62.9 (8.7)	< 0.0001
Median	63	62	0.0002
Min–Max	51–85	36–85	–
Age group, years, *n* (%)			–
18–34	0 (0.0)	0 (0.0)	
35–44	0 (0.0)	1 (0.6)	
45–54	10 (5.1)	18 (11.4)	
55–64	110 (56.1)	104 (65.8)	
≥ 65	76 (38.8)	35 (22.2)	
Geographic region, *n* (%)			0.18
Northeast	42 (21.4)	29 (18.4)	
Midwest	62 (31.6)	37 (23.4)	
South	66 (33.7)	63 (39.9)	
West	26 (13.3)	29 (18.4)	
Unknown	0 (0.0)	0 (0.0)	
Insurance type on index claim, *n* (%)			–
Commercial	95 (48.5)	88 (55.7)	
Medicaid	1 (0.5)	4 (2.5)	
Medicare	77 (39.3)	37 (23.4)	
Self-insured	23 (11.7)	29 (18.4)	
Unknown	0 (0.0)	0 (0.0)	
Health plan type, *n* (%)			–
HMO	65 (33.2)	38 (24.1)	
PPO	113 (57.7)	103 (65.2)	
POS	9 (4.6)	10 (6.3)	
Consumer-directed	5 (2.6)	3 (1.9)	
Indemnity	3 (1.5)	1 (0.6)	
Other	1 (0.5)	3 (1.9)	
Index year, *n* (%)			0.22
2014	41 (20.9)	25 (15.8)	
2015	21 (10.7)	13 (8.2)	
2016	14 (7.1)	14 (8.9)	
2017	23 (11.7)	19 (12.0)	
2018	21 (10.7)	20 (12.7)	
2019	32 (16.3)	26 (16.5)	
2020	19 (9.7)	29 (18.4)	
2021	25 (12.8)	12 (7.6)	
Comorbidities, *n* (%)			
Alcohol/drug abuse	17 (8.7)	24 (15.2)	0.06
Asthma	7 (3.6)	12 (7.6)	0.1
Cardiac arrhythmia	37 (18.9)	29 (18.4)	0.9
Cardiac valvular disease	11 (5.6)	9 (5.7)	0.97
Cerebrovascular disease	10 (5.1)	11 (7.0)	0.46
Chronic kidney disease (excluding end-stage renal failure/dialysis)	23 (11.7)	10 (6.3)	0.08
Chronic pain/fibromyalgia	56 (28.6)	46 (29.1)	0.91
Congestive heart failure	11 (5.6)	12 (7.6)	0.45
COPD	11 (5.6)	11 (7.0)	0.6
Dementia/Alzheimer’s disease	5 (2.6)	0 (0.0)	0.07
Depression	22 (11.2)	24 (15.2)	0.27
Diabetes	45 (23.0)	25 (15.8)	0.09
Dyslipidemia	93 (47.4)	68 (43.0)	0.41
Epilepsy/seizure disorder	1 (0.5)	1 (0.6)	1
Hepatitis	2 (1.0)	3 (1.9)	0.66
HIV/AIDS	4 (2.0)	0 (0.0)	0.13
Hypertension	121 (61.7)	98 (62.0)	0.96
Liver/gallbladder/pancreas disease	33 (16.8)	42 (26.6)	0.03
Myocardial infarction	40 (20.4)	28 (15.8)	0.27
Osteoarthritis	102 (52.0)	88 (55.7)	0.49
Paralysis/hemiplegia/paraplegia	8 (4.1)	2 (1.3)	0.2
Peptic ulcer disease	0 (0.0)	1 (0.6)	0.45
Peripheral vascular disease	24 (12.2)	17 (10.8)	0.66
Renal failure/dialysis	6 (3.1)	2 (1.3)	0.31
Rheumatologic disease	9 (4.6)	6 (3.8)	0.71
Schizophrenia	1 (0.5)	0 (0.0)	1
Sleep disorders	38 (19.4)	27 (17.1)	0.58
Smoking or history of smoking	41 (20.9)	29 (18.4)	0.55
Thyroid disease	12 (6.1)	4 (2.5)	0.11
NCI comorbidity index			
Mean (SD)	1.0 (1.5)	0.9 (1.5)	0.66
Median	0	0	
Min–Max	0–8	0–6	
NCI category, *n* (%)			0.34
0	115 (58.7)	102 (64.6)	
> 0 to < 1	0 (0.0)	0 (0.0)	
1 to < 2	43 (21.9)	23 (14.6)	
2 to < 3	14 (7.1)	14 (8.9)	
> 3	24 (12.2)	19 (12.0)	
PDG score – ICD-9-CM^b^			
Mean (SD)	0.0 (0.2)	0.1 (0.3)	0.51
Median	0	0	
Min–Max	0–1	0–2	
PDG category – ICD-9-CM, *n* (%)^b^			0.78
0	188 (95.9)	150 (94.9)	
> 0 to < 1	0 (0.0)	0 (0.0)	
1 to < 2	8 (4.1)	7 (4.4)	
2 to < 3	0 (0.0)	1 (0.6)	
> 3	0 (0.0)	0 (0.0)	
PDG score – ICD-10-CM^c^			
Mean (SD)	0.3 (0.7)	0.5 (0.8)	0.01
Median	0	0	
Min–Max	0–3	0–3	
PDG category – ICD-10-CM, *n* (%)^c^			0.02
0	158 (80.6)	105 (66.5)	
> 0 to < 1	0 (0.0)	0 (0.0)	
1 to < 2	22 (11.2)	32 (20.3)	
2 to < 3	13 (6.6)	17 (10.8)	
> 3	3 (1.5)	4 (2.5)	

^a^*p* values correspond to comparisons between the 1L ARPI/2L ARPI and 1L ARPI/2L taxane cohorts. Values in bold indicate statistical significance

^b^Calculated as the sum of 12 binary indicators based on claims with ICD-9-CM codes for the following: alcohol disorders, anxiety disorders, bipolar disorders, impulse control, major depression, organic disorders, personality disorders, PTSD, schizophrenic disorders, substance use, other psychotic disorders, other disorders (including atypical depressive disorder, episodic mood disorder not otherwise specified, episodic mood disorder not elsewhere classified, and cyclothymic disorder)

^c^Calculated as the sum of 11 binary indicators based on claims with ICD-10-CM codes for the following: adult personality disorders, anxiety disorders, behavioral syndromes, childhood and adolescence psychiatric disorders, intellectual disabilities, known physiological disorders, mood disorders, pervasive disorders, schizophrenia, substance use, unspecified disorder

1L: first-line; 2L: second-line; AIDS: acquired immunodeficiency syndrome; ARPI: androgen receptor pathway inhibitor; COPD: chronic obstructive pulmonary disease; HIV: human immunodeficiency virus; HMO: health maintenance organization; ICD-9-CM: International Classification of Diseases, 9th Revision, Clinical Modification; ICD-10-CM: International Classification of Diseases, 10th Revision, Clinical Modification; Max: maximum; Min: minimum; NCI: National Cancer Institute; PDG: Psychiatric Diagnosis Group; POS: point of service; PPO: preferred provider organization; PTSD: post-traumatic stress disorder; SD: standard deviation

### Early discontinuation

**Fig. 1:**
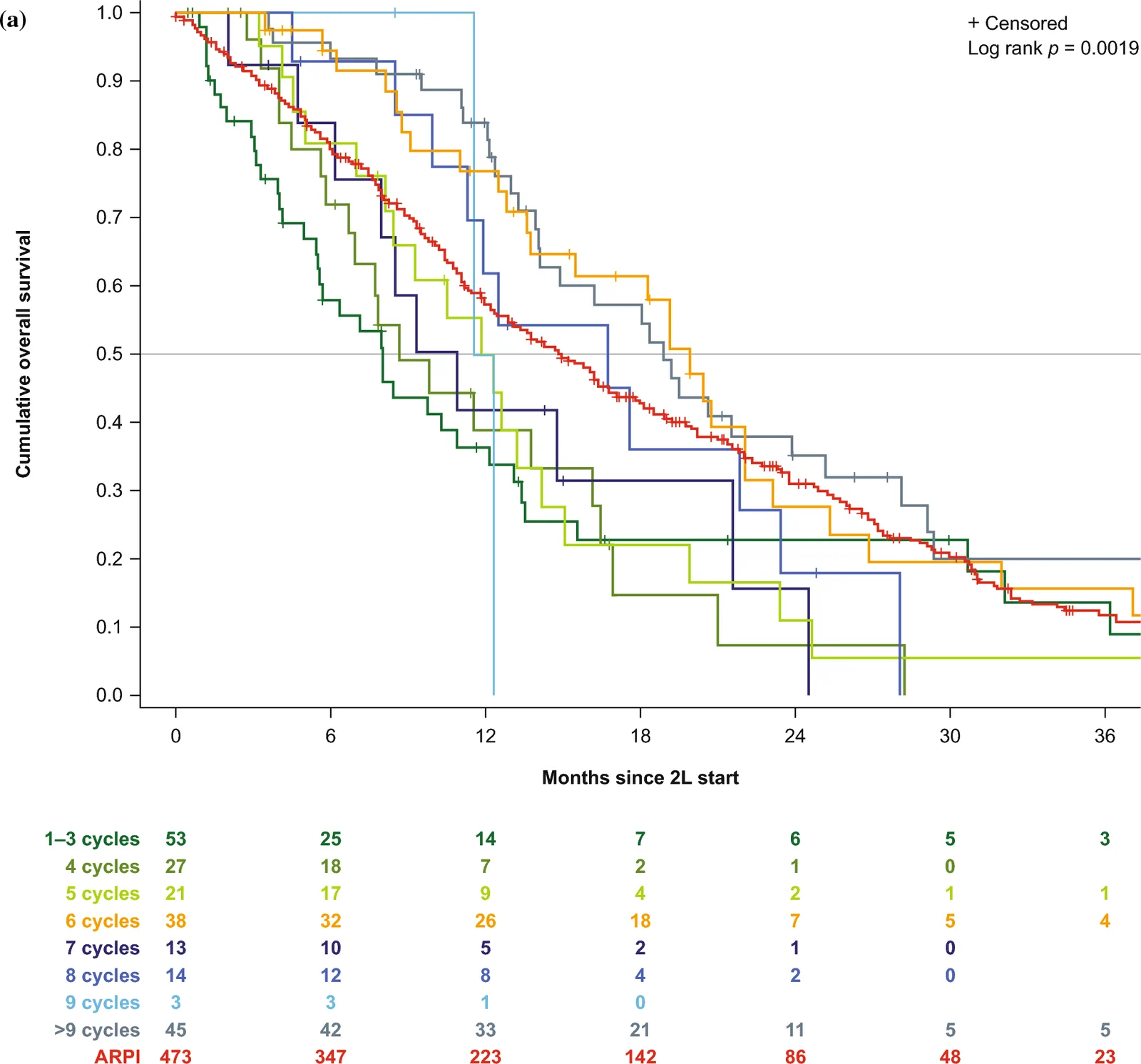

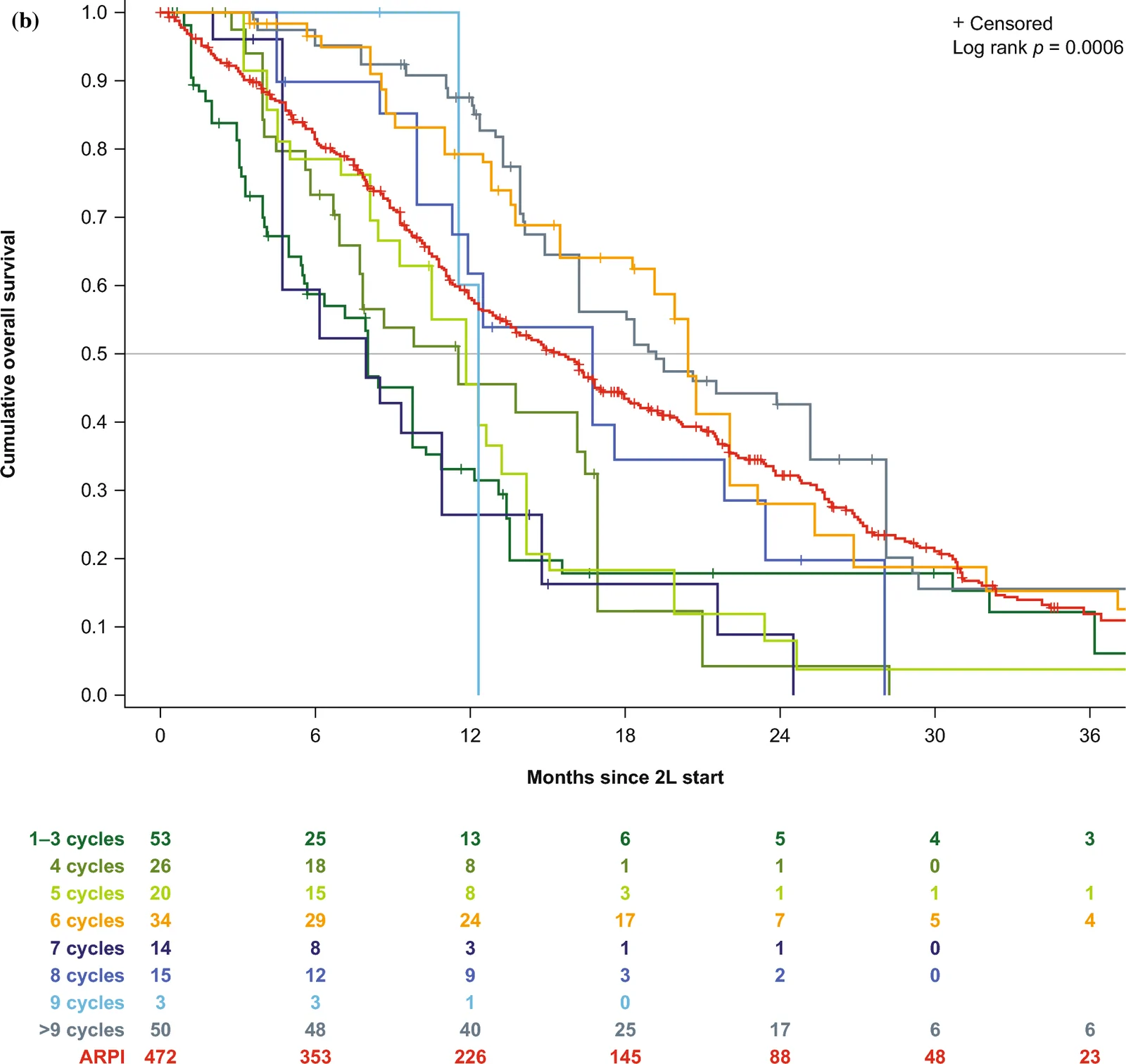
Cumulative OS in the 2L taxane group, by number of taxane cycles, compared with the 2L ARPI group—unadjusted (a) and adjusted for known confounders (b) IPTW was used to adjust for known potential confounders. OS was calculated as the time from the start of 2L therapy to mortality. If mortality was not reached, patients were censored at the latest of the last oral start date, last visit date, or last clinic note date, or if they initiated a clinical study drug at any time. The number of patients at risk at each time point is shown below the graph.2L: second-line; ARPI: androgen receptor pathway inhibitor; IPTW: inverse probability of treatment weighting; OS: overall survival

**Fig. 2:**
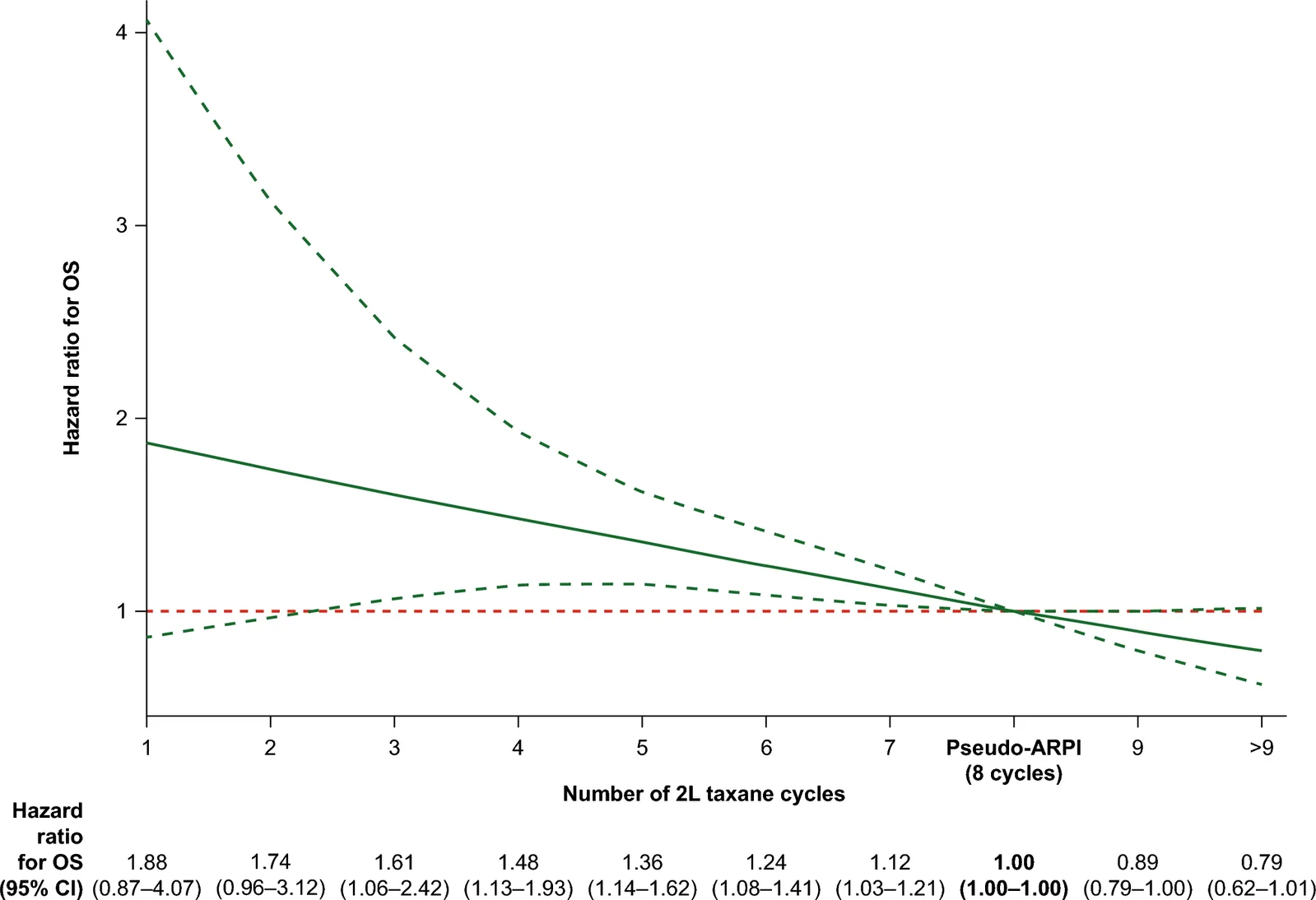
Hazard ratio for OS by number of taxane cycles vs. the pseudo-ARPI (8 taxane cycles) HR < 1 signifies a decreased risk of mortality compared with the reference group (pseudo-ARPI: 8 cycles of taxanes). 2L: second-line; ARPI: androgen receptor pathway inhibitor; CI: confidence interval; HR: hazard ratio; OS: overall survival

Patients in the 2L taxane group of the Flatiron cohort had a median of 6 taxane cycles, over a median duration of 98 days. Patients in the 2L taxane group of the PharMetrics cohort had a median of 4 taxane cycles, over a median duration of 138 days. Using the HR for death (Table 5) and the median OS (Fig. 1), the number of 2L taxane cycles associated with comparable OS to the 2L ARPI group was determined to be 8 in both the unadjusted and adjusted analyses. This threshold was supported by the results of the OS spline analysis (Fig. 2). Thus, the threshold for ‘early discontinuation’ was established as 8 cycles.

**Table 5:** HR for death in the 2L taxane group, by number of taxane cycles, compared with the 2L ARPI group^a^

Treatment	Median OS (IQR)	HR (95% CI)
Unadjusted		
2L ARPI	14.88 (12.91–16.66)	REFERENCE
2L taxane		
1–3 cycles	8.05 (5.45–12.16)	1.58 (1.01–2.45)
4 cycles	8.71 (6.70–16.13)	1.86 (1.27–2.74)
5 cycles	11.86 (8.11–14.19)	1.53 (0.99–2.35)
6 cycles	19.91 (13.57–22.08)	0.81 (0.57–1.13)
7 cycles	10.91 (4.73–21.62)	1.60 (0.96–2.67)
8 cycles	16.76 (9.92–23.43)	1.13 (0.74–1.71)
9 cycles	11.93 (11.53–NE)	1.47 (0.83–2.62)
> 9 cycles	18.89 (14.03–25.17)	0.74 (0.54–1.01)
IPTW-adjusted^b^		
2L ARPI	15.41 (13.21–17.02)	REFERENCE
2L taxane		
1–3 cycles	8.05 (4.14–10.91)	1.43 (0.77–2.66)
4 cycles	11.53 (5.78–16.95)	1.67 (0.98–2.85)
5 cycles	11.86 (7.00–14.19)	1.83 (1.11–3.02)
6 cycles	20.47 (13.73–22.08)	0.80 (0.55–1.18)
7 cycles	7.95 (2.07–14.75)	1.58 (0.65–3.84)
8 cycles	16.76 (4.50–23.43)	1.11 (0.61–2.05)
9 cycles	12.32 (11.53–12.32)	1.35 (0.57–3.23)
> 9 cycles	19.19 (13.96–28.09)	0.74 (0.50–1.10)

^a^The threshold for early discontinuation (8 cycles) is shown in bold. Kaplan–Meier analyses showed patients receiving

> 8 cycles of 2L taxane therapy had numerically longer median PFS and OS vs. patients receiving ≤ 8 cycles of 2L taxane

^b^IPTW was used to adjust for known potential confounders. OS was calculated as the time from the start of 2L therapy to mortality. If mortality was not reached, patients were censored at the latest of the last oral start date, last visit date, or last clinic note date, or if they initiated a clinical study drug at any time

2L: second-line; ARPI: androgen receptor pathway inhibitor; CI: confidence interval; HR: hazard ratio; IPTW: inverse probability of treatment weighting; IQR: interquartile range; NE: not estimable; OS: overall survival; PFS: progression-free survival

Among the 214 patients in the Flatiron cohort receiving 2L taxane therapy, 166 (77.6%) received ≤ 8 cycles and were considered early discontinuers. The remaining 48 (22.4%) received > 8 cycles. Among the 158 patients in the PharMetrics cohort receiving 2L taxane therapy, 138 (87.3%) received ≤ 8 cycles and were thus considered early discontinuers. The remaining 20 (12.7%) received > 8 cycles.

### PFS

**Fig. 3:**
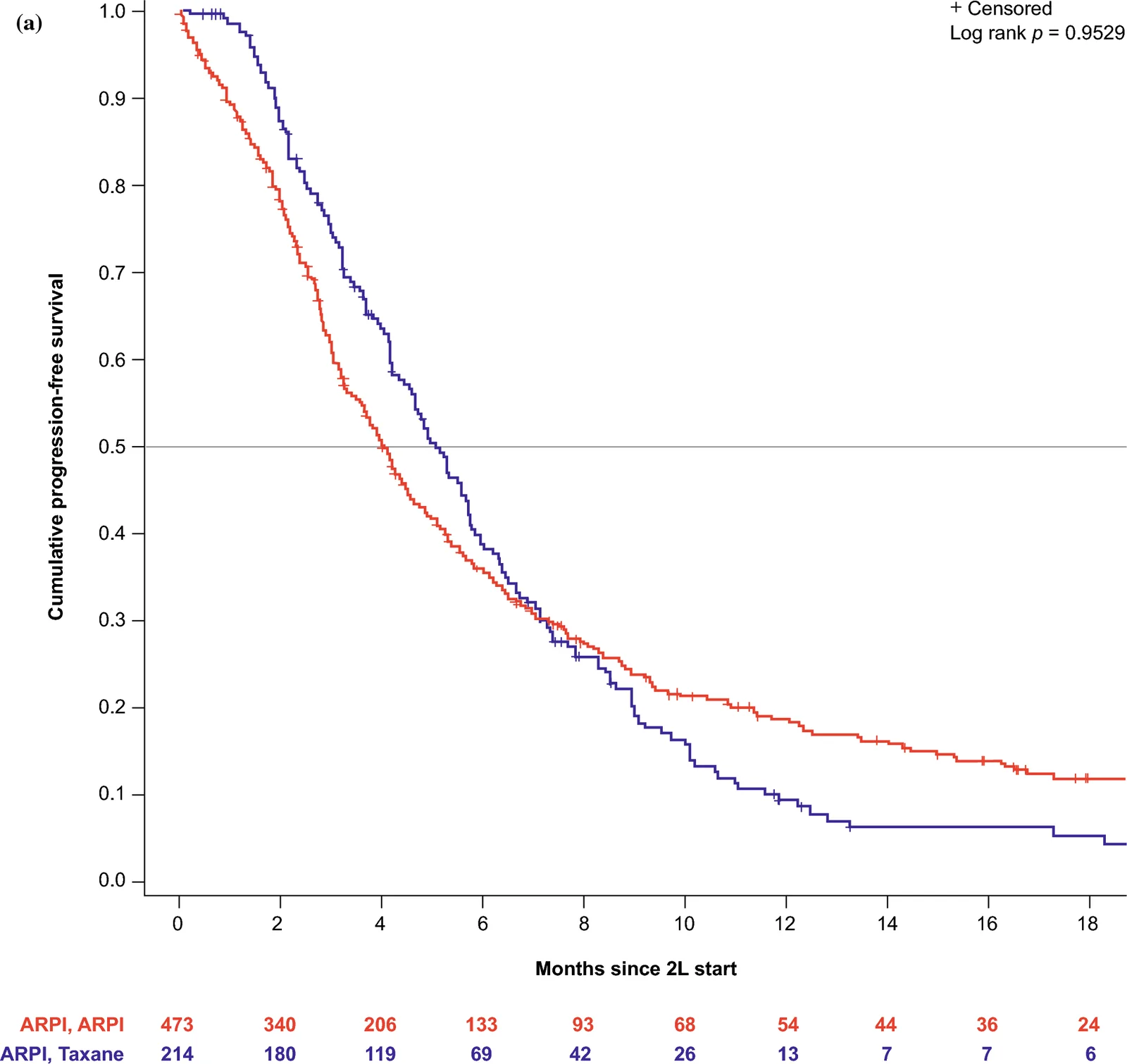

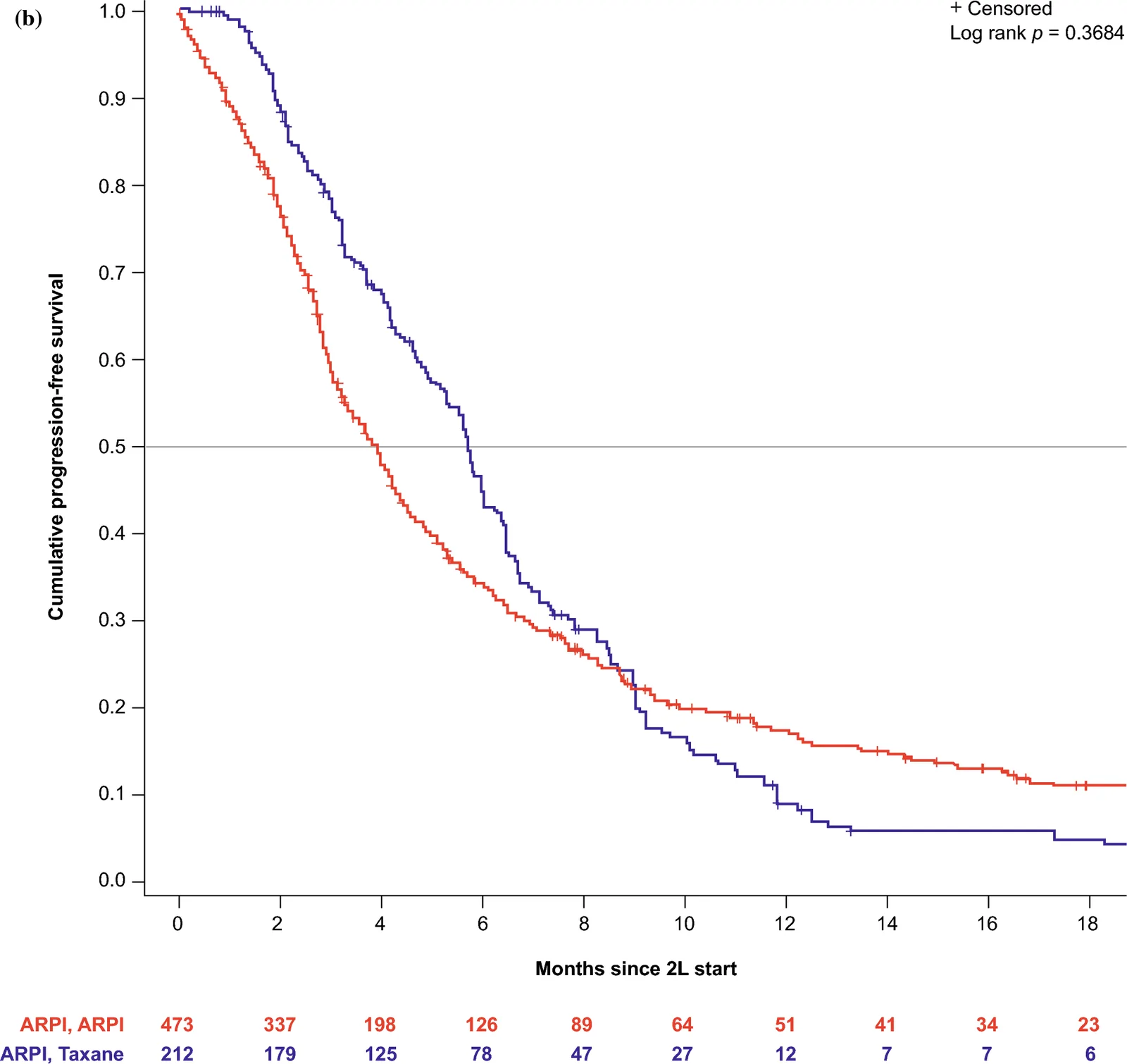

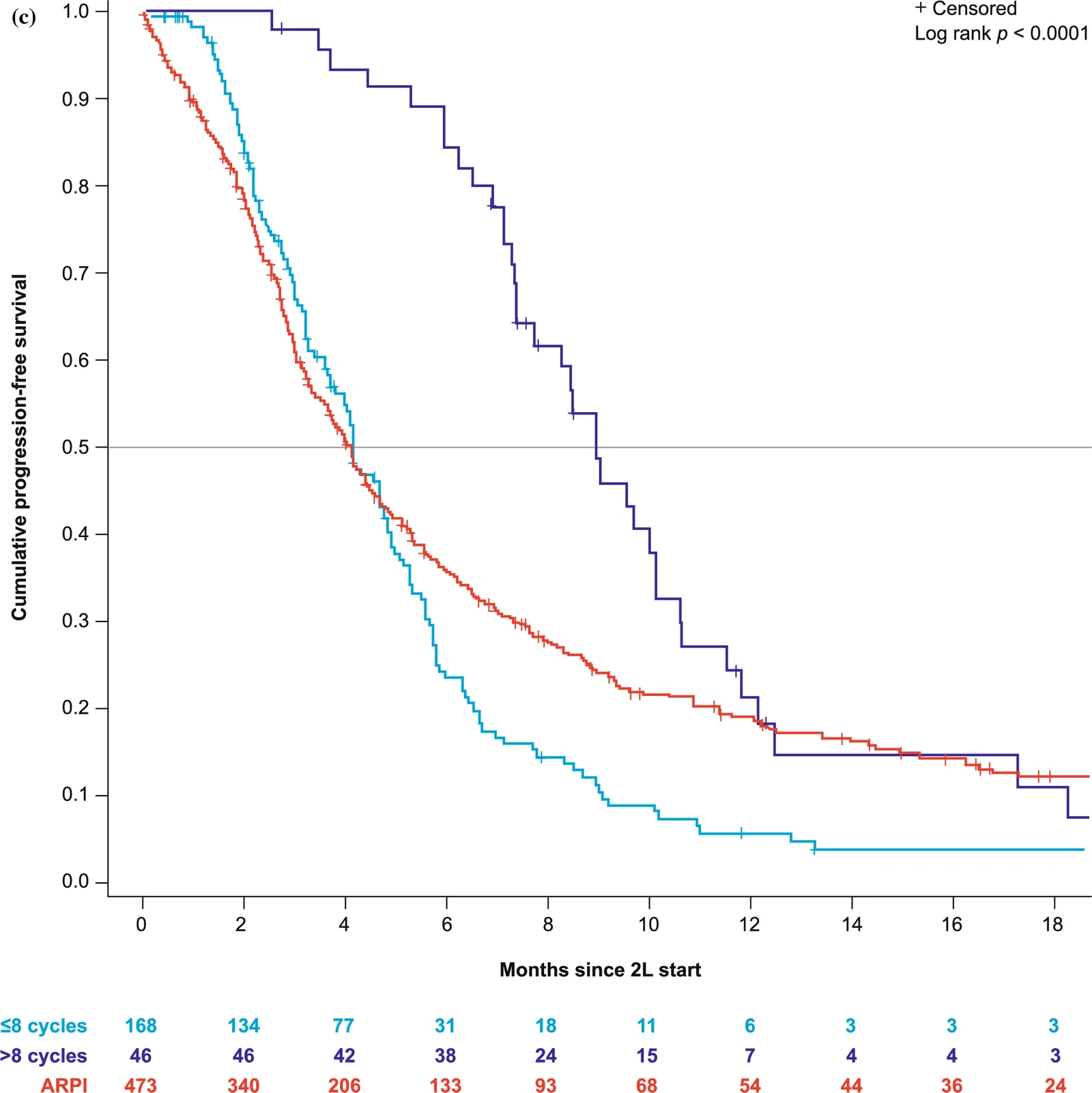

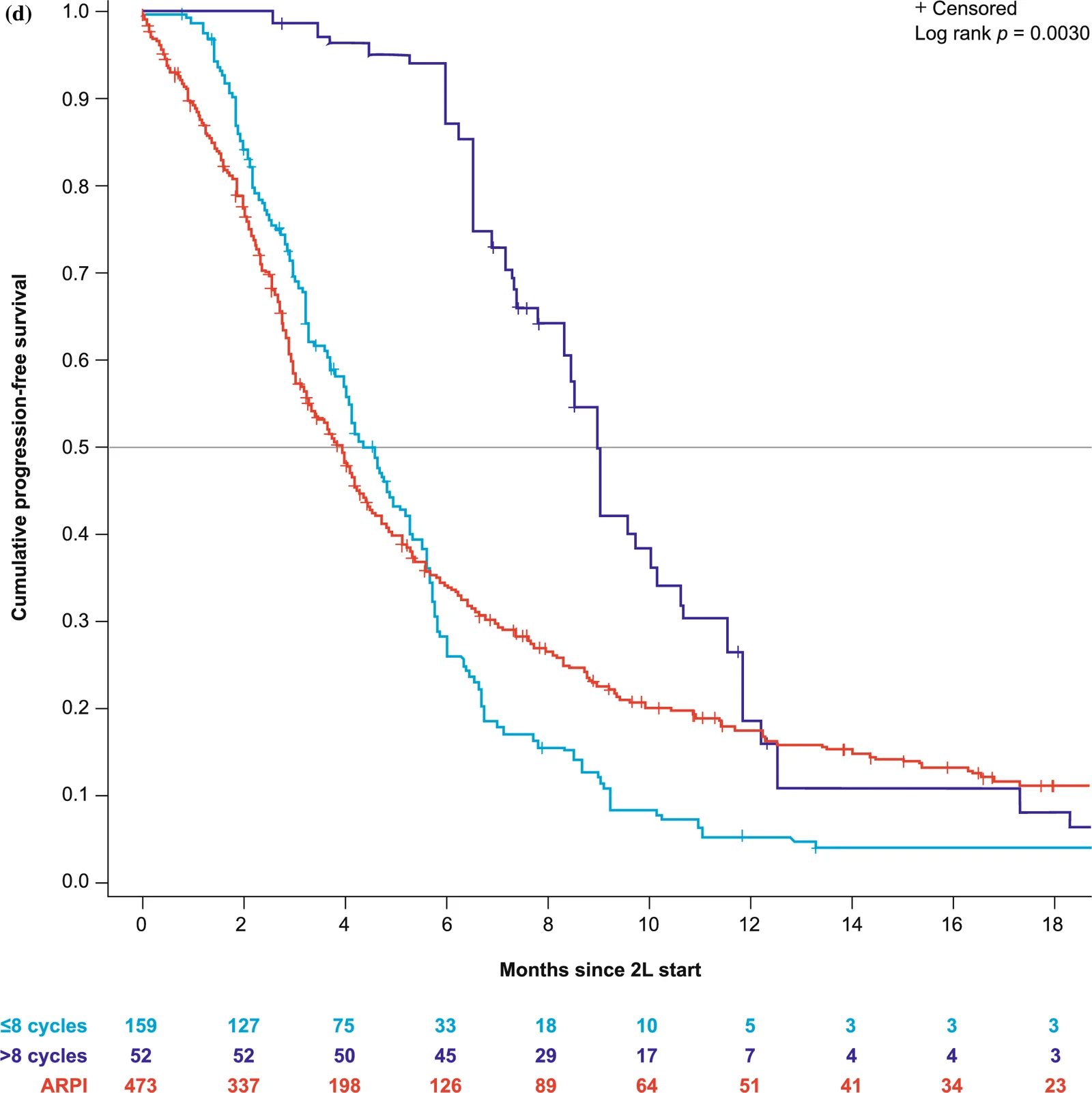
Cumulative PFS in the 2L taxane group versus the 2L ARPI group: overall—unadjusted (a) and adjusted for known confounders (b) and in patients with > 8 or ≤ 8 cycles – unadjusted (c) and adjusted (d) IPTW was used to adjust for known potential confounders. PFS was calculated as the time from the start of 2L therapy to the earliest of progression or mortality before the last known clinical activity, defined as the last clinic note date. If neither progression nor mortality was reached, patients were censored at the last clinic note date or if they initiated a clinical study drug at any time. The number of patients at risk at each time point is shown below the graph. 2L: second-line; ARPI: androgen receptor pathway inhibitor; IPTW: inverse probability of treatment weighting; PFS: progression-free survival

In the unadjusted analyses, 2L taxane therapy was associated with numerically longer median PFS than 2L ARPI (5.1 vs. 4.1 months; Fig. 3A). Similar results were observed after adjusting for patient characteristics (5.7 vs. 3.9 months; Fig. 3B).

When patients receiving 2L taxane therapy were divided using the threshold for early discontinuation, those receiving > 8 taxane cycles had longer unadjusted median PFS than those receiving ≤ 8 taxane cycles and those receiving 2L ARPIs (9.0 vs. 4.4 vs. 4.1 months; *p* < 0.0001; Fig. 3C). Similarly, when adjusted using IPTW, patients receiving > 8 taxane cycles at 2L had longer median PFS compared with those receiving ≤ 8 taxane cycles as well as those receiving 2L ARPIs (9.0 vs. 4.3 vs. 3.9 months; *p* = 0.0030; Fig. 3D).

### OS

**Fig. 4:**
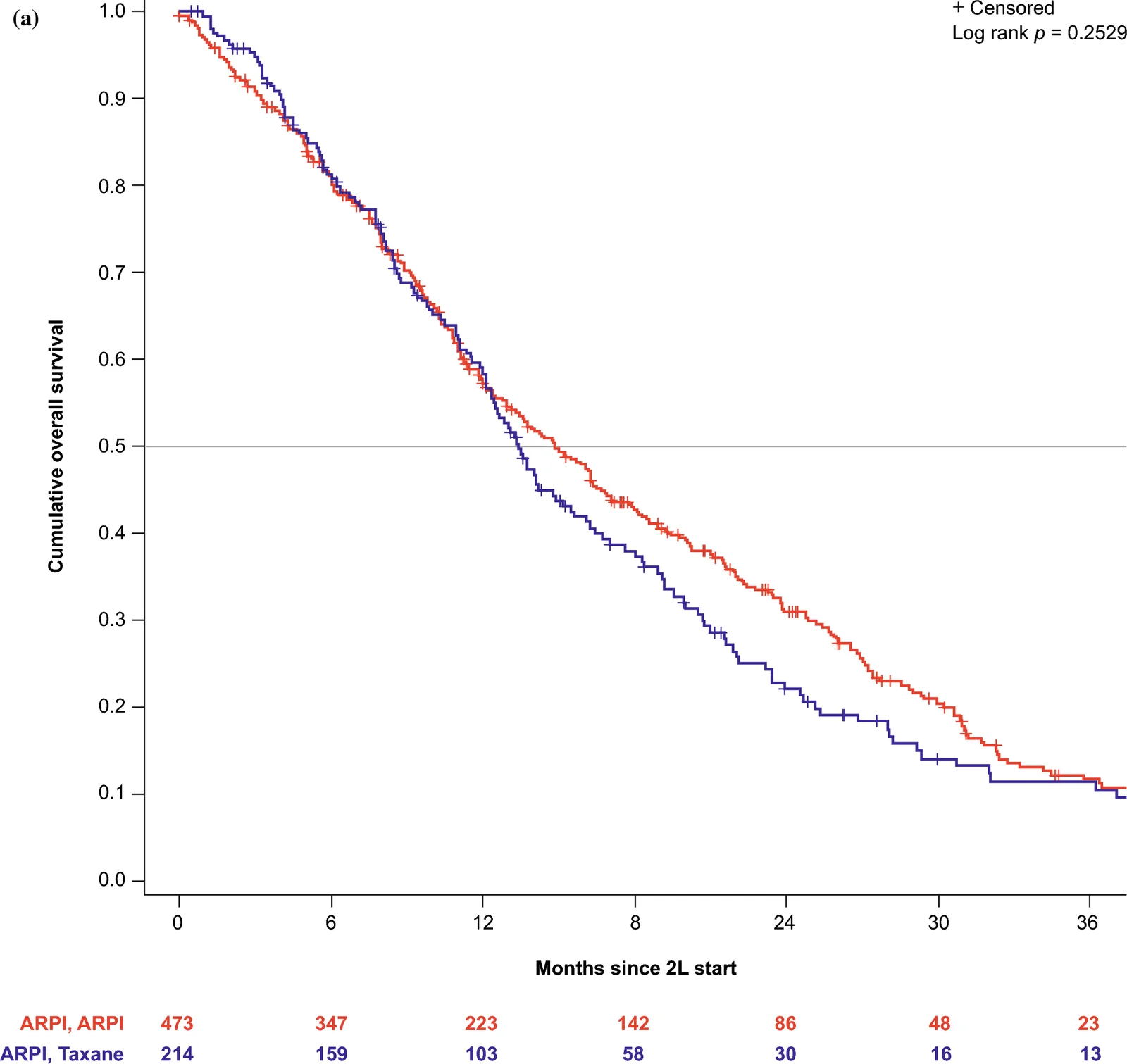

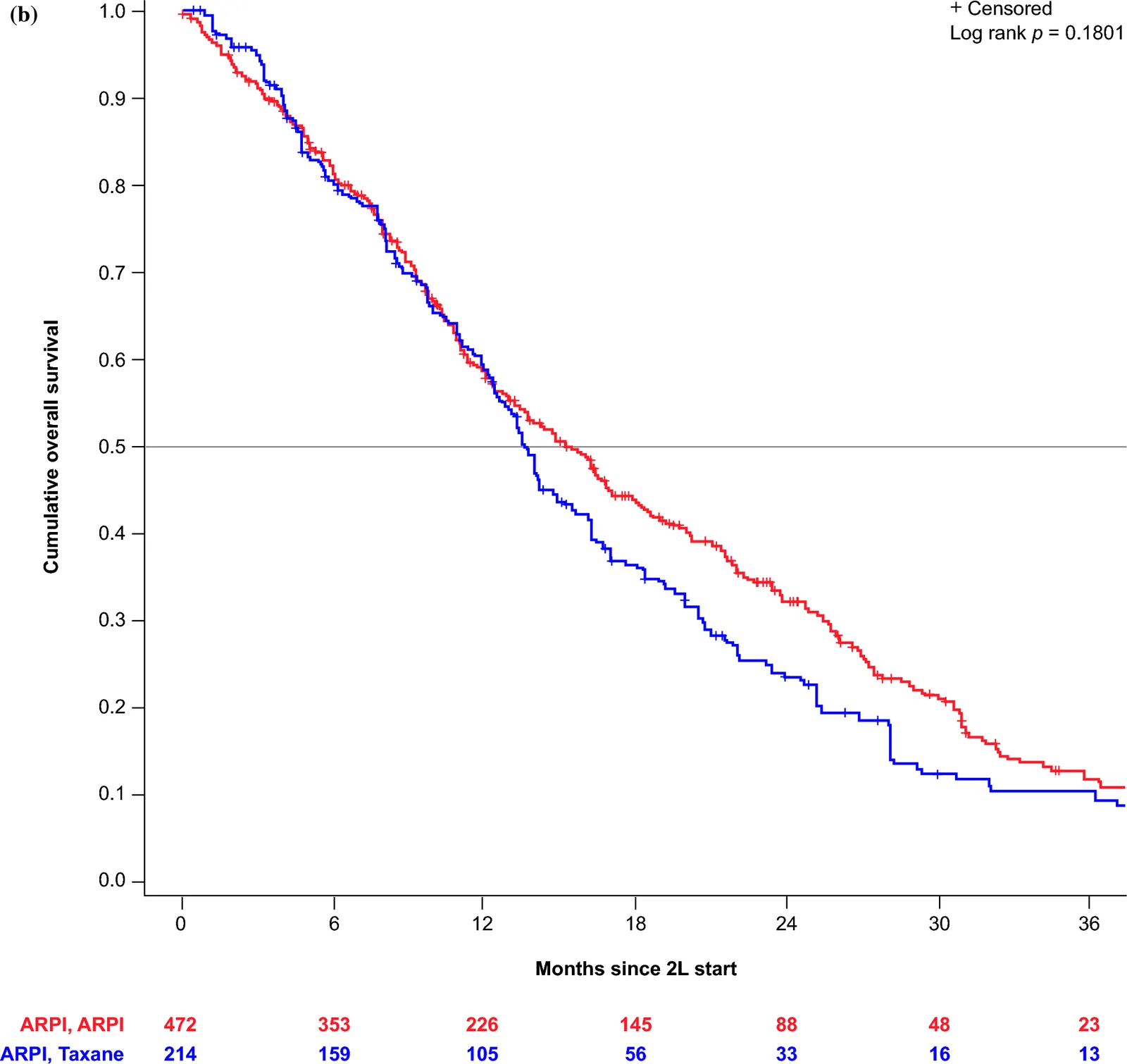

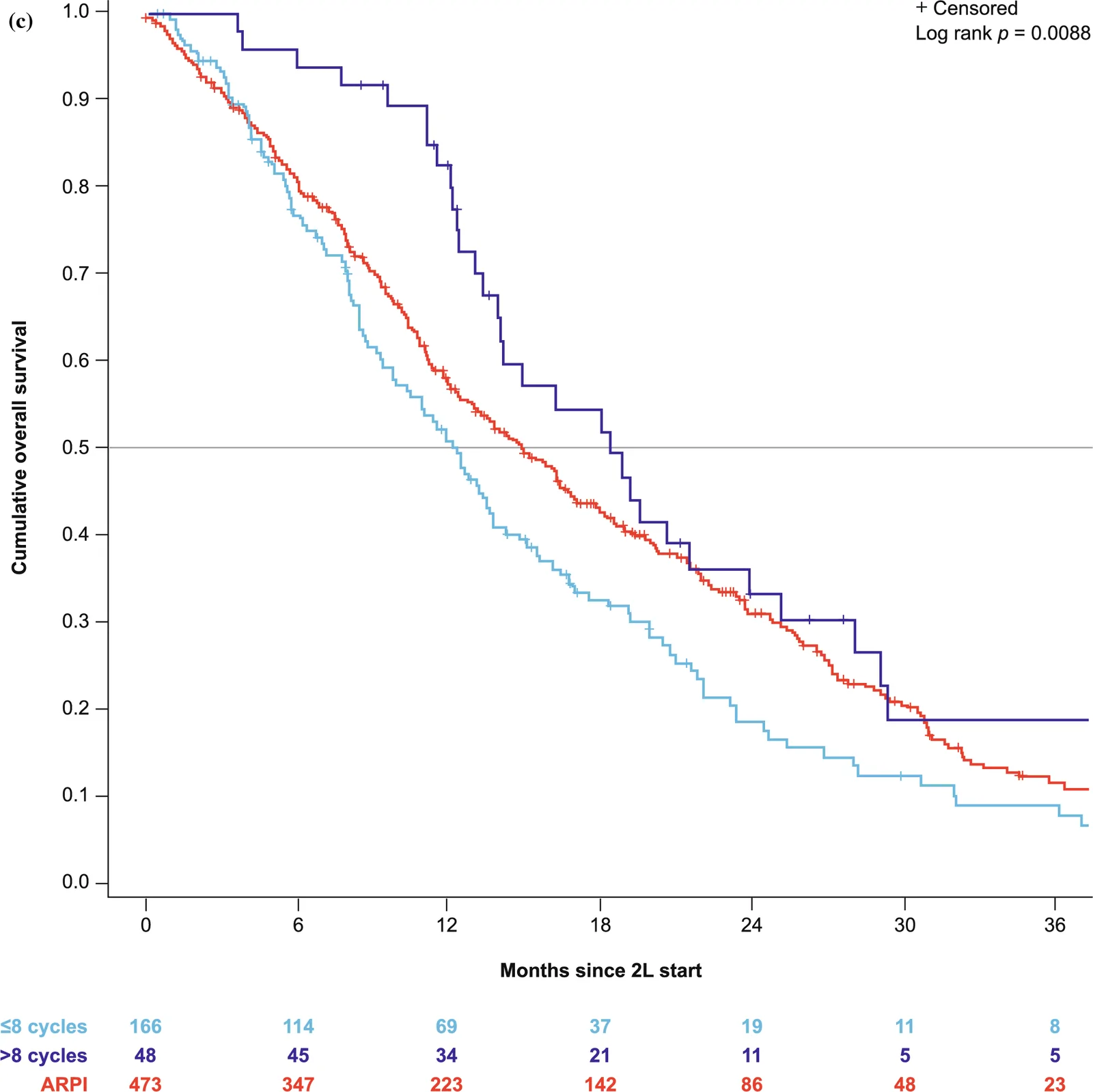

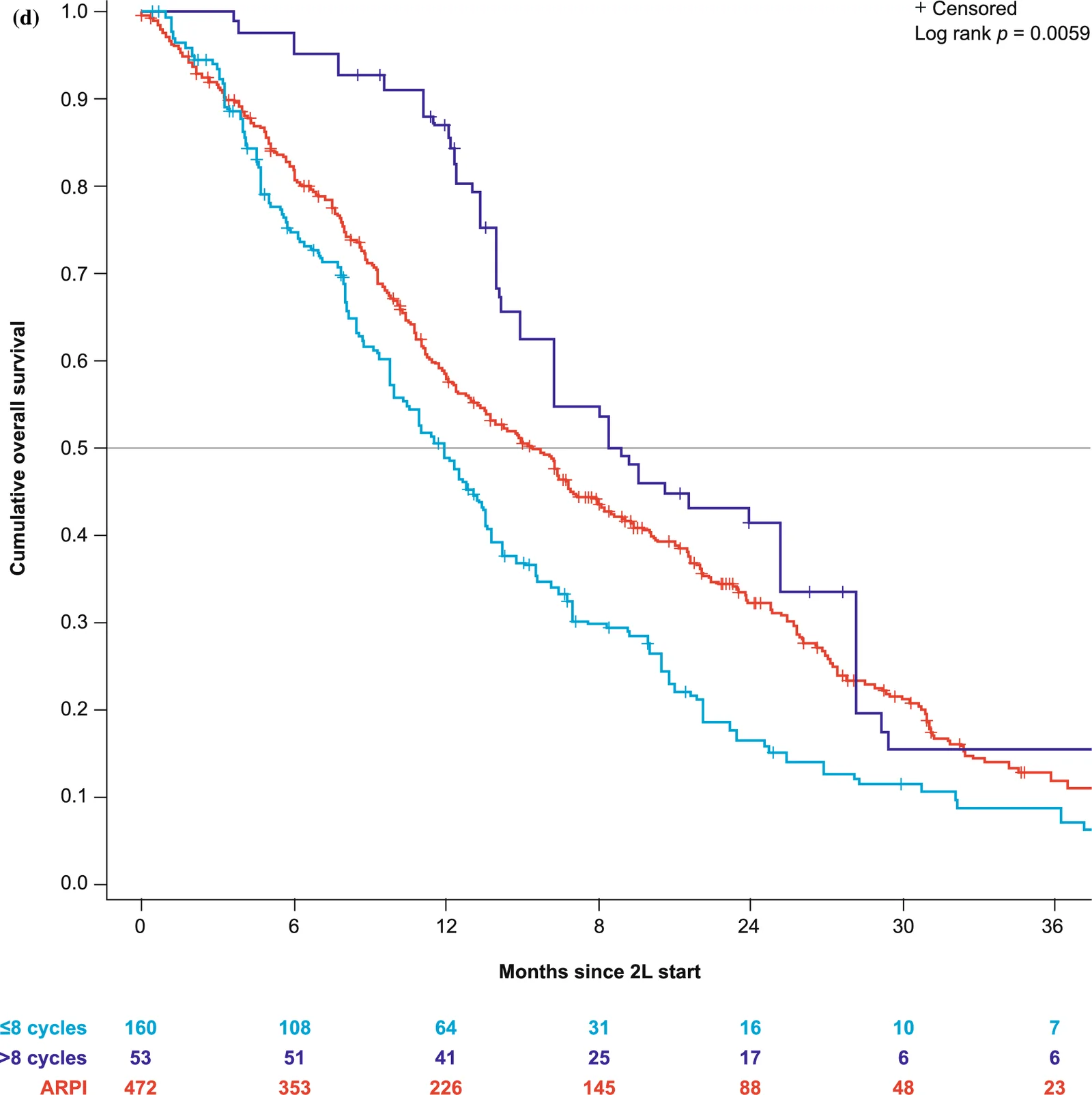
Cumulative OS in the 2L taxane group versus the 2L ARPI group: overall—unadjusted (a) and adjusted for known confounders (b) and in patients with > 8 or ≤ 8 cycles—unadjusted (c) and adjusted (d) IPTW was used to adjust for known potential confounders. OS was calculated as the time from the start of 2L therapy to mortality. If mortality was not reached, patients were censored at the latest of the last oral start date, last visit date, or last clinic note date, or if they initiated a clinical study drug at any time. The number of patients at risk at each time point is shown below the graph. 2L: second-line; ARPI: androgen receptor pathway inhibitor; IPTW: inverse probability of treatment weighting; OS: overall survival.`

In the unadjusted analyses, 2L taxane therapy was associated with numerically shorter median OS than 2L ARPI therapy (13.4 vs. 14.9 months; Fig. 4A). Similar results were observed in the adjusted analyses (13.6 vs. 15.4 months; Fig. 4B).

Patients receiving > 8 taxane cycles in 2L had longer unadjusted median OS than those receiving ≤ 8 taxane cycles and those receiving ARPIs (18.4 vs. 12.3 vs. 14.9 months; *p* = 0.0088; Fig. 4C). After adjustment, patients receiving > 8 cycles still had a longer median OS compared with those receiving ≤ 8 cycles as well as those receiving ARPIs (18.4 vs. 11.9 vs. 15.4 months; *p* = 0.0059; Fig. 4D).

### AEs and AE-associated healthcare costs

Most patients in the PharMetrics Plus 2L taxane group experienced chemotherapy-associated AEs. The most common were blood and lymphatic disorders, infections and infestations, gastrointestinal (GI) disorders, and nervous system disorders (Table 6). More patients who received > 8 taxane cycles experienced AEs compared with those who received ≤ 8 cycles (95.0% vs. 88.4%; *p* = 0.70). However, patients with ≤ 8 cycles had higher AE-related total healthcare costs compared with those who did not ($6,334 vs. $3,429 PPPM; *p* = 0.11).

**Table 6:** Taxane-associated AEs and related costs in patients who did and did not discontinue 2L taxane therapy early

	2L taxane (> 8 cycles) *N* = 20	2L taxane (≤ 8 cycles) *N* = 138	*p* value^a^
AEs			
Any AE, *n* (%)	19 (95.0)	122 (88.4)	0.70
Blood and lymphatic disorders	14 (70.0)	91 (65.9)	0.72
Gastrointestinal disorders	10 (50.0)	69 (50.0)	1.00
Bleeding events	1 (5.0)	22 (15.9)	0.31
Infections and infestations	16 (80.0)	66 (47.8)	0.01
Nervous system disorders	10 (50.0)	48 (34.8)	0.19
Skin and subcutaneous tissue disorders	3 (15.0)	4 (2.9)	0.04
Vascular disorders	1 (5.0)	10 (7.2)	1.00
Renal disorders	3 (15.0)	23 (16.7)	1.00
Liver disorders	1 (5.0)	6 (4.3)	1.00
Cardiovascular disorders	7 (35.0)	32 (23.2)	0.27
Other unspecified AEs of antineoplastics	6 (30.0)	18 (13.0)	0.09
Number of unique types of AEs			
Mean (SD)	3.6 (1.8)	2.8 (1.8)	0.08
Median	3	3	0.09
Min–Max	0–8	0–7	
Number of unique types of AEs, *n* (%)			
0	1 (5.0)	16 (11.6)	0.70
1–2	3 (15.0)	48 (34.8)	0.08
3–4	9 (45.0)	45 (32.6)	0.27
≥ 5	7 (35.0)	29 (21.0)	0.17
AE-related healthcare costs, PPPM^b^			
Total costs			
Mean (SD)	$3,429 ($5,526)	$6,334 ($7,850)	0.11
Median	$571	$3,918	0.06
Medical costs			
Mean (SD)	$3,410 ($5,531)	$6,017 ($7,819)	0.15
Median	$568	$3,662	0.08
Pharmacy costs			
Mean (SD)	$583 ($2,487)	$485 ($2,058)	0.85
Median	$0	$0	0.59

AEs and AE-associated healthcare costs based on threshold of early discontinuation of 2L taxane treatment, defined

≤ 8 cycles (i.e., claims) of the index taxane. AE-related claims were defined as claims with diagnosis, procedure, or drug codes for any of the included AEs

^a^*p* values correspond to comparisons within the 1L ARPI/2L taxane cohort between patients who discontinued 2L taxane treatment early and those who did not. Values in bold indicate statistical significance

^b^Measured from the date of initiation of 2L therapy (index date) until the end of the 2L taxane treatment

1L: first-line; 2L: second-line; AE: adverse event; ARPI: androgen receptor pathway inhibitor; Max: maximum; Min: minimum; PPPM: per patient per month; SD: standard deviation

## Discussion

The NCCN Guidelines favor 2L taxane-based chemotherapy over switching to a second ARPI in pre-taxane, post-ARPI patients with mCRPC [21]. Despite this, real-world evidence, including these results, has shown that ARPIs are the most common treatment in 2L [20].

Using a combination of statistical approaches, the number of 2L taxane cycles with the most comparable clinical outcomes to 2L ARPI was determined to be 8. Overall, only 22% of patients in the Flatiron cohort and 13% in the PharMetrics cohort received > 8 cycles of taxanes, while 78% and 87%, respectively, were early discontinuers. Early discontinuers had shorter PFS and OS than patients who received > 8 cycles of taxane and those who received ARPIs at 2L. The median PFS and OS for early discontinuers were shorter for patients receiving > 8 cycles (4.3 vs. 9.0 months and 11.9 vs. 18.4 months), which is consistent with prior real-world studies showing that more taxane cycles are associated with longer OS. Additionally, early discontinuers were slightly less likely to experience AEs (88% vs. 95%) but had mean AE-related healthcare costs that (though non-significant) were almost twice as high as patients who did not discontinue early ($6,334 vs. $3,429 PPPM).

In this study, patients received a median of 4–6 cycles of 2L taxane therapy after treatment with 1L ARPI, which aligns with the authors’ own clinical experience. It also falls within the 3–12 cycles reported in previous real-world analyses, suggesting that patients continue to receive considerably fewer than the 10 cycles of taxanes aimed for in pivotal clinical trials [9, 27–30]. Prior studies have also divided patients receiving taxanes into two groups depending on a cut-off for the number of cycles received [28, 31–41]. However, the selection of cut-offs in these studies is mostly unclear; some have appeared to use the median number of cycles (usually 6), some the target number of 10 cycles in the registrational trial protocols, and others an arbitrary number (8 or 9 cycles). Overall, results have consistently shown that more cycles are associated with better survival outcomes and that most patients discontinue treatment before the cut-off point.

We defined the threshold for early discontinuation of taxanes by comparison with ARPIs – the most common type of 2L therapy for mCRPC in the real-world setting [20, 23]. This novel approach allowed us to ground our taxane cut-off in terms of impact on real-world clinical outcomes and comparison with the most relevant comparator treatment. To help ensure the robustness of the analyses, Kaplan–Meier curves, IPTW balancing, and spline curves were used to examine the data in different ways.

Although previous retrospective analyses have shown that 2L taxane therapy may be more beneficial than a second ARPI in patients who have received 1L ARPI, this study highlights the importance of the number of taxane cycles that patients receive [42–45].

Early discontinuation of taxanes is most often due to disease progression and/or AEs, followed by patient request and physician decision [46–48]. Taxanes are associated with a range of toxicities including myelosuppression, neuropathy, hypersensitivity reactions, GI bleeding, and infections [25]. Skin and subcutaneous tissue disorders and infections and infestations were more common in patients receiving > 8 taxane cycles in our study. Dermatological AEs are common with taxane therapy and are dose-dependent [49], possibly explaining the increase in dermatological AEs seen in patients receiving > 8 taxane cycles. The increase in infections and the numerically greater proportion of patients with ≥ 3 unique types of AEs in patients receiving > 8 taxane cycles could also be attributed to cumulative toxicity [40, 50]. Here, AEs were observed in almost 90% of early discontinuers, and their AE-related costs were > $6,000 PPPM, suggesting that AEs are one likely explanation for the high rates of early discontinuation. Previous studies have shown that patients treated in community clinics have lower rates of taxane utilization [20, 51]. Oral therapies may be preferred in community settings due to easier administration and better tolerability than taxanes [20]. Travel time to treatment centers can also determine the use of specialist healthcare services, such as taxane therapy [52]. Clinical characteristics, including ECOG PS, age, frailty, and comorbidities are also important considerations in treatment decision-making [51, 53], as patients in poorer overall health are more likely to experience AEs and toxicity [54, 55]. Finally, there is a significant cost associated with the treatment of mCRPC [56], in particular intravenous taxane therapy [51, 57]. Collectively, these reasons may lead to selection of alternative therapies, dose reductions or early discontinuation when taxanes are selected [57]. Since the end of the study period, there have been several advances in treatments. These include the US Food and Drug Administration (FDA) approvals of poly ADP-ribose polymerase (PARP) inhibitors for certain patients [58] and the US FDA approval of Lutetium-177 (^177^Lu)–PSMA-617 for the treatment of patients with prostate-specific membrane antigen-positive mCRPC who have already received an ARPI and taxane-based chemotherapy [59] as well as for those who are considered appropriate to delay taxane-based chemotherapy and received a prior ARPI [60].

### Limitations

We attempted to improve upon prior studies by providing rational and data-driven support for the cut-off used to define early discontinuation. Multiple analytic methods were employed to ensure robustness and support 8 cycles as a meaningful cut-off and the best definition for this analysis. Further confirmation for this cut-off using other datasets and methods is warranted. While inherent bias was addressed as far as possible, patients receiving more cycles may represent a subgroup with more favorable underlying prognosis. Although IPTW was used to adjust for known potential confounders, other potential confounding variables and measures of disease severity and frailty such as bone pain intensity, nutritional status, and geriatric assessment were unknown. Future studies with other study designs, such as prospective studies or landmark analyses, to address treatment selection and immortal time biases are welcome.

Major limitations include the unmeasured and uncontrollable differences between the Flatiron Health and IQVIA PharMetrics Plus databases. These differences impose limitations on the conclusions and inferences that can be made from the evidence when presented together. Additionally, as the specific 2L taxanes that patients received were grouped together for the analyses, the impact of any differences between these agents could not be evaluated. Some patients treated with cabazitaxel may have previously received docetaxel; the impact of this on the number of cycles of cabazitaxel received was not evaluated. Similarly, the contribution of each ARPI treatment to the analyses was not assessed. Furthermore, as neither database contains information as to why treatment was discontinued, the study could not distinguish between various reasons for stopping, e.g. disease progression or toxicity. Flatiron Health EHR data are obtained primarily through participating community and academic oncology centers; clinical management that occurs outside of participating centers is not captured. As with any database study, misclassification and incomplete data entry are possible. Similarly, for the PharMetrics Plus administrative healthcare claims, there are limitations inherent to the use of any claims data, such as coding errors or omissions, coding for rule-out rather than actual disease, and the lack of possibility to verify reported diagnoses. Additionally, PharMetrics Plus is primarily a commercial claims database with under-representation of patients aged > 65 years. Finally, data may not be generalizable to other populations beyond those analyzed. Future analyses should assess whether the results presented here hold true for different subsets of patients, such as those with high volume disease or liver metastases.

## Conclusions

The real-world use of taxanes is hindered by AEs, which can lead to early discontinuation and suboptimal outcomes in patients with mCRPC who had received 1L ARPIs. Overall, 2L taxane therapy was associated with similar median PFS and shorter OS. However, most patients in this study received ≤ 8 cycles of taxane at 2L, most likely due to AEs or progression, which was associated with shorter PFS and OS than either > 8 cycles of taxane or 2L ARPI. In addition, patients who received ≤ 8 cycles of taxane therapy had higher AE-related costs than patients who received > 8 cycles. Nonetheless, results suggest that more effective and better tolerated therapies are needed for this patient population.

## Supplementary Information

Below is the link to the electronic supplementary material.

Supplementary file1 (DOCX 47 kb)

## Data Availability

This study was conducted using data from IQVIA and Flatiron. The IQVIA data are proprietary and were used under license. They are not publicly available. Additionally, this study used de-identified data provided by Flatiron Health, Inc. The data are not publicly available due to contractual and privacy restrictions.
