# Correction to: Real-world clinical outcomes and economic burden of discontinuation of taxane therapy among patients with metastatic castration-resistant prostate cancer

**DOI:** 10.1007/s10147-026-03194-w

**Published:** 2026-09-30

**Authors:** Neal Shore, Jennifer Nguyen, Barinder Kang, Amrita Sawhney, Clare Byrne, Jackson Tang, Magdaliz Gorritz, Chi-Chang Chen, Jeetvan Patel

**Affiliations:** 1 START Carolinas/Carolina Urologic Research Center, Myrtle Beach, SC USA; 2 Novartis Pharmaceuticals Corporation, East Hanover, NJ USA; 3 Asclepius Analytics, New York, NY USA; 4 IQVIA, Wayne, PA USA

**Correction to: International Journal of Clinical Oncology (2026) 31:1451–1471**. **https://doi.org/10.1007/s10147-026-03102-2**.

In the original version of this article, the given name of Jennifer Nguyen was inadvertently omitted. The name was displayed correctly in all versions at the time of publication.

Given Name: Jennifer

Family Name: Nguyen

The original article has been corrected.

