# Supplementary material for: Real-world clinical outcomes and economic burden of discontinuation of taxane therapy among patients with metastatic castration-resistant prostate cancer
Source: Int J Clin Oncol. 2026 Jun 25;31(8):1451–71. doi: 10.1007/s10147-026-03102-2 (PMC13401553; doi:10.1007/s10147-026-03102-2)
Supplement: Supplementary file 1 — Supplementary file1 (DOCX 47 kb) [file 10147_2026_3102_MOESM1_ESM.docx]

**Supplemental methods**

**Inclusion criteria (Flatiron Health database)**

- Confirmed diagnosis of PC (ICD-9: 185.x; ICD-10: C61.x)
- Confirmed castration resistance, defined by Flatiron Health as any of the following in the unstructured elements of the patient’s EHR:
  - Explicit documentation of CRPC by the physician (CRPC diagnosis, hormone resistant/refractory, androgen insensitive/independent, etc.)
  - Rising PSA level of ≥2 ng/mL after initial hormone therapy, followed by at least one further rise ≤3 months after the ﬁrst increase
  - Physician documentation of a rising PSA level or PSA progression on the 1L of hormone therapy, accompanied by a change in treatment
  - In instances where multiple sources of evidence are available, the earliest time point will be recorded as the date of CRPC diagnosis
- Explicit documentation of metastatic disease by the physician; histology confirmed as adenocarcinoma or not otherwise specified (NOS)
- Two or more documented clinical visits on different days
- At least 18 years of age at index
- Male sex
- Receipt of at least one of the following therapies post-mCRPC diagnosis: abiraterone, enzalutamide, docetaxel, cabazitaxel, sipuleucel-T, radium-223, and mitoxantrone on or after Jul 1, 2013 and on or before to Dec 31, 2019
- History of treatment with one ARPI (abiraterone or enzalutamide) as 1L therapy post-mCRPC diagnosis
- Receipt of either another ARPI (abiraterone or enzalutamide) or a taxane (docetaxel or cabazitaxel) as 2L therapy
- Included patients must switch from the first ARPI to a second ARPI or taxane directly, with no other treatment lines in between

**Exclusion criteria (Flatiron Health database)**

- Unclear castration resistance status or no date of onset of resistance
- Diagnosis of a second primary cancer or secondary cancer prior to index date

**Inclusion criteria (IQVIA PharMetrics^®^ Plus database)**

- Patients with ≥2 outpatient claims, at least 30 days apart, or one inpatient claim with a diagnosis of PC (ICD-9-CM: 185; ICD-10-CM: C61) at any time during the index period
- Patients with ≥2 outpatient claims, at least 30 days apart, or one inpatient claim with a diagnosis of metastasis (ICD-9-CM codes: 196.*, 197.*, 198.*; ICD-10-CM codes: C77.*, C78.*, C79.*) at any time occurring on or after the PC diagnosis date
- Patients with evidence of medical or surgical castration (before or after date of metastasis), defined as:
  - Surgical – at least one claim with any of the following codes: CPT: 54520, 54530, 54535, 54690, 56318; ICD-9-CM procedure: 62.4, 62.41, 62.42; ICD-9-CM diagnosis: V45.77; ICD-10-CM procedure: 0VBC0ZZ, 0VBC3ZZ, 0VBC4ZZ, 0VTC0ZZ, 0VTC4ZZ; ICD-10-CM diagnosis: Z90.79, Z19.2
  - Medical – at least one claim for any of the following drugs: anti-androgens (bicalutamide, flutamide, nilutamide, enzalutamide, apalutamide, darolutamide), androgen synthesis inhibitor (degarelix, abiraterone), estrogens (DES), or progestins (megestrol); AND
    ≥1 claim for any of the following drugs: LHRH antagonists (goserelin, histrelin, leuprolide, triptorelin)
- Male sex
- At least 18 years of age at index
- Receipt of at least one of the following therapies post-mCRPC diagnosis: docetaxel, cabazitaxel, abiraterone, enzalutamide, radium-223, mitoxantrone, estramustine, sipuleucel-T
- History of treatment with one ARPI (abiraterone or enzalutamide) as 1L therapy post-mCRPC diagnosis
- Receipt of either another ARPI (abiraterone or enzalutamide) or a taxane (docetaxel or cabazitaxel)
  - Included patients must switch from the first ARPI to a second ARPI or taxane directly, with no other treatment lines in between
- At least 12 months of pre-index continuous enrollment with medical and pharmacy benefits
- At least 1 month of post-index continuous enrollment with medical and pharmacy benefits

**Exclusion criteria (IQVIA PharMetrics^®^ Plus database)**

- Multiple primary tumor types, except non-malignant skin cancer, observed during the 12 months pre-index or any time after index
- Data quality issues, including missing or invalid age, sex, or enrollment dates; Medicare Cost coverage, State Children's Health Insurance Program, or pharmacy-only coverage; or age ≥65 years and not covered by Medicare Advantage

**Statistical analysis**

Analyses for the study were performed using SAS version 9.4 (SAS Institute Inc., Cary, NC, USA) and/or Stata/SE 16.1 (StataCorp LLC, College Station, TX, USA). All patient characteristics and outcomes in both cohorts were summarized descriptively. Continuous variables were described using mean and standard deviation (if normally distributed) or median and interquartile range (if skewed). Categorical variables were described by the number and percentage of patients in each category. The student t-test or Kruskal-Wallis test was used to compare continuous variables between treatment groups. Comparison of categorical data was performed using the Chi-square test, or the Fisher’s exact test if there was an absolute count of <10.

**Statistical methods used for determining the cut-off number of cycles**

First, Kaplan–Meier survival curves for PFS and OS were constructed for the Flatiron Health 2L taxane group stratified by the total number of cycles of taxane received, and the curves were overlaid with survival curves for the 2L ARPI group. These curves demonstrated the absolute probability of PFS and OS for each cycle of taxane, relative to the absolute probability of PFS and OS post-treatment with ARPI. This step was done once for PFS and OS, unadjusted for known potential confounders, and repeated with IPTW to adjust for known potential confounders. For the IPTW approach, propensity scores were generated for the number of cycles of taxane received by patients. The propensity for each number of taxane cycles was calculated with the use of all available patient and disease characteristics.

As a next step, the number of 2L taxane cycles a patient would need to achieve comparable PFS and OS to that observed with 2L ARPI was determined. This number of cycles was then used as a proxy for ARPI in the subsequent analyses to determine the threshold for early discontinuation. Early discontinuers were defined as patients who stopped receiving 2L taxane therapy – for any reason – before the point at which they would have achieved equivalent benefit to patients receiving 2L ARPIs. To accomplish this, Cox regression modeling was used and two items were considered simultaneously: the HR comparing the number of taxane cycles with ARPI that was closest to 1, and the median time to PFS or OS for each taxane cycle that was closest with the median time for ARPI, in which both the hazard ratio (HR) comparing the number of taxane cycles with ARPI that was closest to 1, and the median OS closest to ARPI were determined to inform the definition of the threshold for early discontinuation. Bar graphs were also generated for the HRs for progression and death for each cycle of 2L taxane compared with 2L ARPI. This step was done once for PFS and OS, unadjusted for known potential confounders, and repeated with IPTW to adjust for known potential confounders.

In addition, the effect of each 2L taxane cycle (modeled as a continuous variable transformed with a restricted cubic spline) was compared with the taxane–ARPI equivalency point group (identified in the first secondary objective) as the referent category. Graphs were generated of the HRs for progression and death for each cycle of 2L taxane compared with the pseudo-ARPI, separately for PFS and OS.

**Supplemental Table 1.** Outcomes of interest analyzed per database.

|  | **Effectiveness**  **Flatiron Health EHR** | **Safety and economic analyses**  **IQVIA PharMetrics Plus claims** |
| --- | --- | --- |
| **Primary endpoints** | Patient demographics and clinical characteristics   - Age - Race/ethnicity - ECOG performance status - Gleason score - ALP - PSA - Hb - LDH | Patient demographics and clinical characteristics   - Age - Geographic region - Insurance type - Health plan type - PDG score - NCI Comorbidity Index - Comorbidities |
|  | Treatment patterns   - Total number of taxane cycles - Duration of therapy - Number of patients who progressed during taxane therapy - Number of taxane cycles pre-progression - Number of patients advancing the 3L therapy - Type of 3L therapy | Treatment patterns   - Total number of taxane cycles - Duration of 2L taxane therapy |
| **Secondary endpoints** | Clinical outcomes   - PFS - OS | AEs |
|  |  | Number of unique types of AEs |
|  |  | AE-related total cost |

2L: second-line; 3L: third-line; AE: adverse event; ALP: alkaline phosphatase; ECOG: Eastern Cooperative Oncology Group; EHR: electronic health record; Hb: hemoglobin; LDH: lactate dehydrogenase; NCI: National Cancer Institute; OS: overall survival; PDG: Psychiatric Diagnosis Group; PFS: progression-free survival; PSA: prostate-specific antigen.
